# Prevalence of Talon cusp in Indian population

**DOI:** 10.4317/jced.50650

**Published:** 2012-02-01

**Authors:** Rachana V. Prabhu, Prasanna K. Rao, KM Veena, Prathima Shetty, Laxmikanth Chatra, Prashanth Shenai

**Affiliations:** 1 Reader, Department of Oral Medicine and Radiology, Yenepoya Dental College, Yenepoya University, Mangalore, Karnataka, India; 2 Professor, Department of Oral Medicine and Radiology, Yenepoya Dental College, Yenepoya University, Mangalore, Karnataka, India; 3 Assistant Professor, Department of Oral Medicine and Radiology, Yenepoya Dental College, Yenepoya University, Mangalore, Karnataka, India; 4 Senior Professor and Head, Department of Oral Medicine and Radiology, Yenepoya Dental College, Yenepoya University, Mangalore, Karnataka, India; 5 Senior Professor, Department of Oral Medicine and Radiology, Yenepoya Dental College, Yenepoya University, Mangalore, Karnataka, India

## Abstract

Aim: To investigate the prevalence of the talon cusps in a sample of Indian dental patients and their distribution among different types of teeth. To determine the presence of other dental anomalies associated with the talon cusps. Method: 2740 out patients (1523 males and 1217 females) attending Oral Medicine department from November 2010 to January 2011 were screened for the presence of talon cusps and were subjected to Intra Oral Peri-apical (IOPA) radiograph to rule out any associated anomalies or peri-apical changes. Results: Talon cusps were detected in 16 out of 2740 patients (person prevalence 0.58%). Thirty one teeth were found to have talon cusp. Maxillary lateral incisors were the most commonly affected teeth (54.8%, 17 teeth), followed by maxillary central incisors and canines (16.12%, 5 teeth).Talon cusp was found in two mandibular central incisors (6.45%) and one each in mandibular second and third molar (3.22% each). Seventeen teeth in 7 patients (54.83%) were found to be associated with anomalies like dens invagination (6 teeth, 19.35%), impacted 13, 23 (6 teeth, 19.35%), partial anodontia (3 teeth, 9.67%), geographic and fissured tongue (2 teeth, 6.45%). Peri-apical granuloma was found in one tooth with talon cusp associated with dens invaginatus. None of the patients were found to be associated with any syndromes. Conclusion: Attention should be paid to the presence of the talon cusp and the associated anomalies. Early diagnosis of the talon cusp can help the clinician in preventing the further complications.

** Key words:**Orthopantomography, atheroma, stroke.

## Introduction

A talon cusp (dens evaginatus of anterior tooth) is a well delineated additional cusp located on the surface of an anterior tooth and extends at least half the distance from the cementoenamel junction (CEJ) to the incisal edge (1). The name reflects the resemblance of the most extreme cases to an eagle’s talon when viewed from the occlusal edge. It was first described by Mitchell as an accessory cusp in 1892 and in 1970 it was termed as Talon cusp (1). Various other terms have been used to describe this trait including dens evaginatus, supernumary cusp, horn, hyperplastic cingulum, evaginated odontome, cusped cingulum, accessory cusp and supernumary lingual tubercle (2).

Three fourths of all reported cases are located in the permanent dentition. The cusps predominantly occur on permanent maxillary lateral (55%) or central (33%) incisors and less frequently on mandibular incisors (6%) and maxillary canine (4%) (3).

It is an autosomal dominant condition arising during the morpho-differentiation phase of tooth development. A multifactorial etiology including both genetic and environmental factors has been proposed. An aberrant hy-perproductivity of the dental lamina has also been found to be responsible for its occurance (4,5). The accessory cusp has been seen in association with other dental anomalies like supernumary teeth, odontomas, impacted teeth, peg shaped lateral incisors, dens invaginatus. Talon cusps have been seen in patients with Mohr, Rubinstein Taybi and Sturge – Weber syndromes, Ellis van Creveld syndrome (6). The clinical problems associated with the presence of talon cusps include stagnation of the food, caries, periapical lesions, irritation of tongue during speech and mastication, other soft tissue irritation, compromised esthetics, occlusal interference which may lead to accidental cusp fracture, displacement of the affected tooth, temporomandibular joint pain and periodontal problems because of excessive occlusal force (7).

Extensive prevalence studies have not been performed but estimates suggest the frequency of talon cusp in the population ranges from less than 1% to 8 % (3). In a review of 108 reported cases of talon cusps as case reports in the literature between the years of 1970 and 1995 the authors indicated that about 7.7% of the cases were in permanent teeth and 20% of them were bilaterally distributed (3). The prevalence of talon cusp was reported as 0.55% in Jordanian permanent teeth (8), 0.6% in a Maxican (9), 2.5% in a Hungarian (10) and 5.2% in a Malaysian population (11). No data were found to address the distribution of talon cusps among different tooth types in Indian population.

The present study was aimed to investigate the prevalence of the talon cusps in a sample of Indian dental patients and their distribution among different types of teeth. To determine the presence of other dental anomalies associated with the talon cusps.

## Material and Methods

A total of 2740 out patients (1523 males and 1217 females) attending Oral Medicine department, Yenepoya University, Mangalore, Karnataka – India, from November 2010 to January 2011 were screened for the presence of talon cusps and other hard tissue and soft tissue anomalies. The study sample presented represents the south Indian population. The age of these patients ranged between 4 to 60 years. The patients with talon cusp were then subjected to Intra Oral Periapical (IOPA) radiograph to rule out the associated anomalies or periapical changes. The patients’ records and radiographs were evaluated and the following variables were studied: age, sex distribution, and distribution of talon cusps in different teeth, surfaces involved, radiographic evidence of pulp extension, and associated dental anomalies and complications.

## Results

Talon cusps were detected in 16 out of 2740 patients (person prevalence 0.58%). The detail data of these patients like the age, gender, teeth involved, surfaces involved and the associated anomalies was recorded ( Table 1).

**Table 1:**
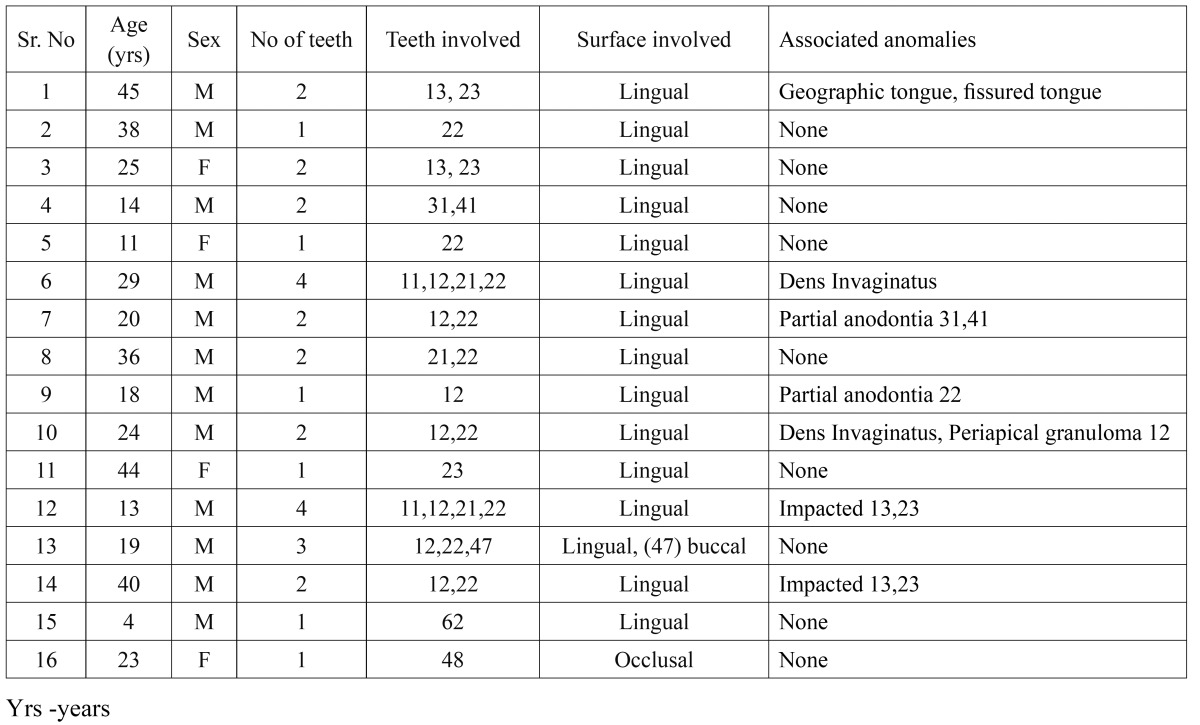
Personal data of 16 patients presenting with the talon cusp.

Thirty one teeth were found to have talon cusp. Out of these 31 affected teeth 96.77 % (30 teeth) were permanent and only one tooth (3.22%) was found to be deciduous. Maxillary teeth (27 teeth, 87.09%) were found to be more involved than the mandibular teeth (4 teeth, 12.90%).

Maxillary lateral incisors were the most commonly affected teeth (54.8%, 17 teeth), followed by maxillary central incisors and canines (16.12%, 5 teeth).Talon cusp was found in two mandibular central incisors (6.45%) and one each in mandibular second and third molar (3.22% each) ( Table 2).

**Table 2:**
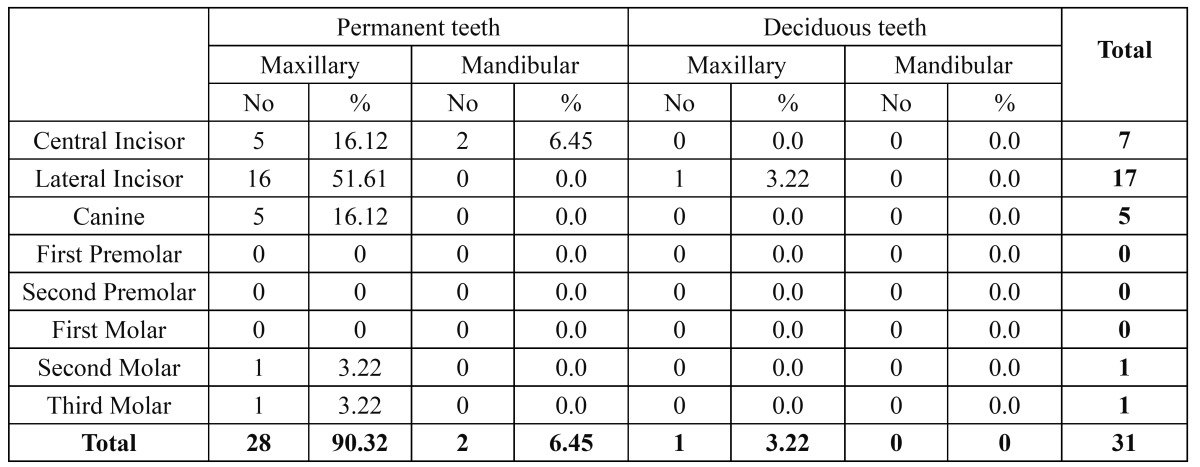
The distribution of talon cusps among different tooth types.

**Figure 1:**
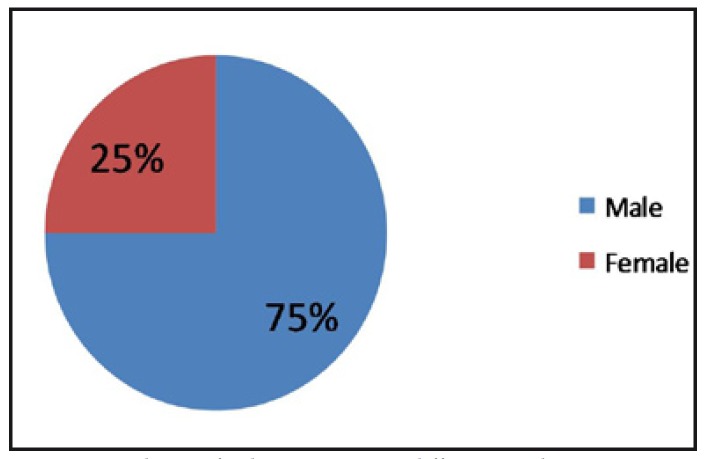
Prevalence of Talon cusp among different gender.

Prevelance of the talon cusp was seen more in males (75%, 12 patients - 26 teeth) than females (25%, 4 patients – 5 teeth) (Fig. 1).

**Figure 2:**
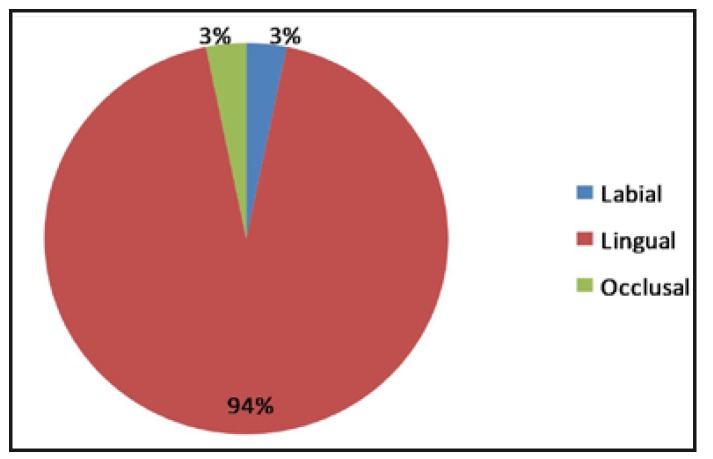
Distribution of the Talon cusp with respect to the surfaces involved.

Talon cusp was found to involve the lingual side more (29 teeth, 94%) than the buccal and occlusal surfaces (3%, 1 tooth each) (Fig. 2).

Bilateral involvement of the talon cusp was seen in 24 teeth (77.41%).

**Figure 3:**
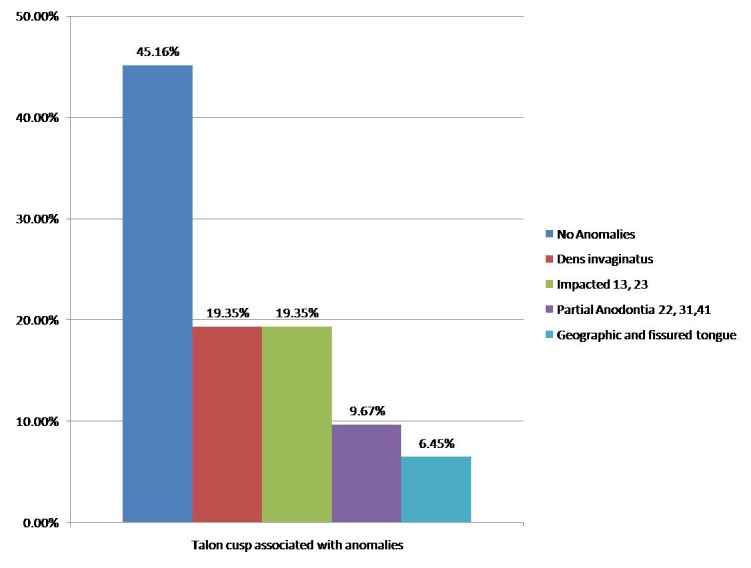
Talon cusp associated with the anomalies.

Seventeen teeth in 7 patients (54.83%) were found to be associated with anomalies like dens invagination (6 teeth in 2 patients, 19.35%), impacted 13, 23 (6 teeth in 2 patients, 19.35%), partial anodontia (3 teeth in 2 patients, 9.67%), geographic and fissured tongue (2 teeth in 1 patient, 6.45%). Peri-apical granuloma was found in one tooth with talon cusp associated with dens invaginatus (Fig. 3).

None of the patients were found to be associated with any syndromes.

## Discussion

There are many cases reported in the literature on talon cusp and their presence with other anomalies. Extensive prevalence studies have not been performed so far. Few prevalence studies done represents Mexican (0.6%) (9), Jordanian (2.4%) (8), Hungarian (2.5%) (10) and Malaysian (5.2%) (11) population. The prevalence of talon cusp in the present study was found to be 0.58%. These results are comparable with what was reported in Mexican population but lower than what was reported in the other nations.

The variation in the talon cusp prevalence could be explained by variation of the condition among different nations or variation in the sample examined or examination criteria.

The occurrence of talon cusps is rare in deciduous teeth. Three fourths of all reported cases are located in the permanent dentition (3). The present study also encountered the prevalence of these cusps to be less (3.22%) in deciduous teeth than in permanent teeth (96.77%).

In the present study maxillary lateral incisors (54.8%) were found to most commonly affected teeth with the condition followed by maxillary central incisors and canines (16.12% each). This is in agreement with the literature (3,12) and the study reported earlier in Turkish (13) population in which 51% of the cases were seen involving lateral incisors. In Jordanian (8) population maxillary canines (46%) were found to be more involved than maxillary lateral and central incisors. The differences could be due to the design of the study or the diagnostic criteria followed. In the present study and the Turkish study diagnosis of the talon cusp is done clinically followed by radiograph for evaluation of the further information where as the Jordanian study is radiographic survey of radiographs from the dental records.

Maxillary teeth are seen to be more affected than the mandibular teeth (3,12,13). In the present study 87.09% of the cases were found to involve the maxillary teeth and only 12.90% were found in the mandibular teeth. Out of which 6.44% were found in mandibular central incisors and 3.22% each in mandibular second and third molar. Mandibular central incisors with talon cusps are found in 3% of reported cases in Turkish population (13). Dens evagination is typically seen on mandibular premolars and rarely has been reported on molars (14). In 2001 Sakiyama (15) reported a case of DE that occurred in a lower second molar. In 1998, Ngeow and Chai (16) reported DE in the wisdom tooth. The reason of less reported cases could be due to the definition criteria. Some researchers point out that the talon cusp should be differentiated from DE whereas other scholars suggest the talon cusp and DE are the same type of anomaly (17).

The cusps are more frequently seen on the lingual aspect. There are some reported cases of talon cusp on the labial aspect (18,19,20) and occlusal aspect of the teeth (14). The present study also found that the distribution of the talon cusp was more on the lingual (93.5%) side than on the buccal and occlusal aspect (3.22% each).

The prevalence of talon cusp was seen more in males (75%) than females (25%) in a ratio of 3:1. This is in accordance with the published data in the literature (8,12).

Bilateral talon cusps were seen in 77.41% of the cases. This also agrees with the data reported in the literature (1,5,6,8,21-25).

The accessory cusp has been seen in association with other dental anomalies like supernumary teeth, odontomas, impacted teeth; peg shaped lateral incisors, dens invaginatus (26). In the present study dens invagination (19.35%) was seen more commonly associated with talon cusp followed by impacted canines (19.35%) and partial anodontia (9.67%). In hong kong children, supernumary teeth and hypodontia was reported in 8.6% of the cases respectively (26). Concomitant occurance of Dens evagination and invagination is relatively rare (27-30).

It is diagnosed only when a radiograph is taken. The line of treatment also varies when concomitant occurance of these two anomalies is seen. One of the patient in the present study presented with the periapical granuloma in a tooth with talon cusp associated with dens invaginatus. It was then treated with root canal treatment. This emphasizes on the early detection of associated anomalies with the talon cusp so if required prophylactic treatment can be undertaken.

6.45% of the cases were found to be associated with geographic and fissured tongue. Developmental anomaly of the tongue associated with the talon cusp has been reported for the first time in the present study. But none of the cases were found to be associated with any known abnormal systemic developmental syndrome.

The present study is the first to report the prevalence of talon cusp in south Indian population. It is the first study to discuss the distribution of the cusps among different tooth types and the associated anomalies in Indian population. The present data will provide more information on the type of the teeth that are more susceptible to this anomaly and the need of the radiographic examination of the affected teeth to avoid the periapical complications. It will also facilitate the understanding of changes in occlusion, periodontal condition and the technical difficulties associated with the endodontic treatment of such teeth.

## References

[1] Mellor JK, Ripa LW (1970). Talon cusp: a clinically significant anomaly. Oral Surg Oral Med Oral Pathol.

[2] Lee CK, King NM, Lo EC, Cho SY (2007). The relationship between a primary maxillary incisor with a talon cusp and the permanent successor: a study of 57 cases. Int J Paediatr Dent.

[3] Dankner E, Harari D, Rotstein I (1996). Dens evaginatus of anterior teeth. Literature review and radiographic survey of 15,000 teeth. Oral Surg Oral Med Oral Pathol Oral Radiol Endod.

[4] Lomcali G, Hazar S, Altinbulak H (1994). Talon cusp: report of five cases. Quintessence Int.

[5] Rantanen AV (1971). Talon cusp. Oral Surg Oral Med Oral Pathol.

[6] Gardener DG, Girgis SS (1979). Talon cusps: a dental anomaly in the Rubinstein–Tyabi syndrome. Oral Surg Oral Med Oral Pathol.

[7] Güngör HC, Altay N, Kaymaz FF (2000). Pulpal tissue in bilateral talon cusps of primary central incisors: report of a case. Oral Surg Oral Med Oral Pathol Oral Radiol Endod.

[8] Hamasha AM, Safadi RA (2010). Prevalence of talon cusps in Jordanian permanent teeth: a radiographic study. BMC Oral Health.

[9] Sedano HO, Carreon Freyre I, Garza de la Garza ML, Gomar Franco CM, Grimaldo Hernandez C, Hernandez Montoya ME (1989). Clinical orodental abnormalities in Mexican children. Oral Surg Oral Med Oral Pathol.

[10] Mavrodisz K, Rozsa N, Budai M, Soos A, Pap I, Tarjan I (2007). Prevalence of accessory tooth cusps in a contemporary and ancestral Hungarian population. Eur J Orthod.

[11] Rusmah Meon (1991). Talon cusp in Malaysia. Aust Dent J.

[12] Omari MA, Hattab FN, Darwazeh AM, Dummer PM (1999). Clinical problems associated with unusual cases of talon cusp. Int Endod J.

[13] Gündüz K, Celenk P (2008). Survey of talon cusps in the permanent dentition of aTurkish population. J Contemp Dent Pract.

[14] Yan-gang R, Li-yang G, Tao HU (2010). Multiple Dens Evaginatus of Premolars and Molars in Chinese Dentition: A Case Report and Literature Review. Int J Oral Sci.

[15] Sakiyama Y (2001). Considerable supplement on the central cusp in the lower second molar. Kaibogaku Zasshi.

[16] Ngeow WC, Chai WL (1998). Dens evaginatus on a wisdom tooth: a diagnostic dilemma. Case report. Aust Dent J.

[17] Levitan ME, Himel VT (2006). Dens evaginatus: literature review, pathophysiology, and comprehensive treatment regimen. J Endod.

[18] Pomeroy E (2009). Labial talon cusps: a South American archaeological case in the deciduous dentition and review of a rare trait. Br Dent J.

[19] Tulunoglu O, Cankala DU, Ozdemir RC (2007). Talon’s cusp: report of four unusual cases. J Indian Soc Pedod Prev Dent.

[20] McNamara T, Haeussler AM, Keane J (1997). Facial talon cusps. Int J Paediatr Dent.

[21] Dayal PK, Mani NJ, Verma PK (1980). Talon cusp: a review and case report. J Dent.

[22] Mader CL (1981). Talon cusp. J Am dent Assoc.

[23] Chen RJ, Chen HS (1986). Talon cusp in primary dentition. Oral Surg Oral Med Oral Pathol.

[24] Davis PJ, Brook AH (1986). The presentation of talon cusp: diagnosis, clinical features, associations, and possible aetiology. Br Dent J.

[25] Salama FS, Hanes CM, Hanes PJ, Ready MA (1990). Talon cusp a review and two case reports on supernumary primary and permanent dentition. J Dent Child.

[26] Cho SY, Ki Y, Chu V, Lee CK (2008). An audit of concomitant dental anomalies with maxillary talon cusps in a group of children from Hong Kong. Prim Dent Care.

[27] Mupparapu M, Singer SR, Goodchild JH (2004). Dens evaginatus and dens invaginatus in a maxillary lateral incisor: Report of a rare occurrence and review of literature. Aust Dent J.

[28] Tiku A, Nadkarni UM, Damle SG (2004). Management of two unusual cases of dens invaginatus and talon cusp associated with other anomalies. J Indian Soc Pedod Prev Dent.

[29] Noikura T, Ooya K, Kikuchi M (1996). Double dens in dente with a central cusp and multituberculism in bilateral maxillary supernumerary central incisors: report of a case. Oral Surg Oral Med Oral Pathol Oral Radiol Endod.

[30] Vardhan TH, Shanmugam S (2010). Dens evaginatus and dens invaginatus in all maxillary incisors: Report of a case. Quintessence Int.

